# Exploring the prebiotic characteristics of crude polysaccharides from coconut testa flour: A comparative analysis of local cultivar

**DOI:** 10.1016/j.heliyon.2024.e30256

**Published:** 2024-04-26

**Authors:** Rasika Gunarathne, Sandani Wijenayake, Chandhi Yalegama, Nazrim Mohammed Marikkar, Jun Lu

**Affiliations:** a Auckland Bioengineering Institute, University of Auckland, Auckland, 1142, New Zealand; b Sabaragamuwa University of Sri Lanka, Belihuloya, Sri Lanka; c Coconut Research Institute of Sri Lanka, Lunuwila, Sri Lanka; d National Institute of Fundamental Studies, Hanthana Road, Kandy, Sri Lanka; e School of Health Science and Engineering, University of Shanghai for Science and Technology, Shanghai, 200093, China; g Maurice Wilkins Centre for Biodiscovery, Auckland, New Zealand; h College of Food Engineering and Nutrition Sciences, Shaanxi Normal University, Xi'an, 710119, Shaanxi Province, China; i College of Food Science and Technology, Nanchang University, Nanchang, 330031, Jiangxi Province, China; j Department of Food and Agriculture Technology, Yangtze Delta Region Institute of Tsinghua University, Zhejiang, Jiaxing, 314006, China

**Keywords:** Coconut testa flour, Crude polysaccharide, FTIR, Prebiotic potential, Probiotic proliferation, *Lactobacillus* sp.

## Abstract

Crude polysaccharides (CPs) of coconut testa flour (CTF) of five local cultivars; Commercial hybrid (COM), Gon Thembili (GT), Ran Thembili (RT), San Ramon (SR), and Tall × Tall (TT) were isolated in this study to examine to evaluate potential prebiotic properties and structural characteristics. The isolated CPs were subjected to FTIR analysis to identify functional groups of biomolecules. Digestibility tests were performed on CPs of different coconut cultivars using artificial human gastric juice. CPs were applied to *Lactobacillus* sp. and *Escherichia coli* to evaluate their proliferation *in vitro*. FTIR results showed that the occurrence of peaks between 1200 cm^−1^ – 1000 cm^−1^ was indicative of the presence of C–O side groups and C–*O*–C glycosidic bonds. CPs of all cultivars displayed strong resistance to hydrolysis (94–97 %) by human gastric juice when compared to inulin and fructooligosaccharides (FOS). The higher proliferation of *Lactobacillus* sp. was detected on all five CPs when compared to that of glucose. The SR cultivar (1.09) showed a higher prebiotic activity score compared to fructooligosaccharides (0.92) and inulin (0.81). These findings suggest that CPs isolated from CTF of local coconut cultivars possess potential prebiotic characteristics that can be used in the preparation of functional foods. Further studies on this aspect are granted to enable the effective use of CTF in promoting human gut health.

## 1 Introduction

Coconut testa (CT), the brown outer covering of the coconut kernel, is essentially removed in the manufacturing of virgin coconut oil, coconut milk, and desiccated coconut as it imparts a brown hue to the oil and diminishes the visual appeal of other products. Regarding the significant production of coconut-based products in Sri Lanka, a substantial amount of coconut testa is generated as a byproduct [1].

Coconut testa flour (CTF) is a substance obtained from the defatted residues of dehydrated coconut testa left after oil extraction. A recent study by Marasinghe et al. [2] showed that coconut testa flour of local cultivars COM, GT, RT, SR, and TT was found to have roughly 2.27–4.27 % moisture, 7.93–23.49 % fat, 23.82–32.22 % protein, and 42.55–59.24 % total carbohydrates. As a source of non-cereal flour, CTF was reported to display some beneficial physical properties, which are of considerable importance to being used as an ingredient in bakery products [3]. A follow-up study by Gunarathne et al. [4] indicated that CTF of all local coconut cultivars was a rich source of phenolics and flavonoids, which had the ability to act as potent antioxidants and anti-hyperglycemic agents. This was in accordance with the previous findings by Adekola and Marikkar [5], who reported on the anti-hyperglycemic effect of coconut testa. These preliminary findings have subsequently paved the way for the development of nutritious cookies [6] and some staple foods [7] using CTF as an ingredient. However, studies directly focusing on the functional food attributes of CTF of Sri Lankan coconut cultivars are scarcely reported.

Functional foods have increasingly become important for human health as they are a substantial source of nutrients that provide benefits beyond their nutritional value while reducing risk factors that lead to chronic ailments. Non-digestible food ingredients are generally said to be prebiotics, which may selectively stimulate the growth and activity of one or more bacterial species present in the colon [8]. Probiotics like *Bifidobacterium* and *Lactobacillus* have been recognized to have the ability to ferment the prebiotics to selectively stimulate the probiotics in the digestive tract. This may result in the stimulation of the immune system, inhibition of pathogen growth, and reduction of blood ammonia and cholesterol levels. Additionally, they might help reduce constipation and infantile diarrhea as well as relieve irritable bowel syndrome. According to previous studies, the majority of prebiotics are plant-based food products. Owing to the health benefits of prebiotics, there is an ever-increasing demand for the discovery of novel prebiotic sources from locally available materials. In a recent report, Khan et al. [9] discussed the innovations in non-dairy-based prebiotics, especially wastes from fruits, green leafy vegetables, legumes, and cereals. Although many studies have been performed targeting several agro-byproducts, the prebiotic potential of crude polysaccharides isolated from CTF of locally grown cultivars has not been previously reported. It is hoped that an initiative on the prebiotic assessment of CTF of different Sri Lankan cultivars will eventually expand their practical uses in the healthcare industry.

## 2 Materials and methods

### 2.1 Materials

Coconuts of twelve-month maturity were collected from five different local cultivars: COM, GT, RT, SR, and TT, maintained at the varietal blocks of Coconut Research Institute, Lunuwila, Sri Lanka. Fifty nuts of each cultivar were sampled for seasoning, followed by de-husking. The preparation of coconut testa flour for analysis was done in accordance with the method previously described by Marasinghe et al. [2]. Briefly, the shells of the nuts were removed manually using hammers, while de-pairing was done using manually operated knives. The fresh testa of individual cultivars was disintegrated separately into medium-sized particles using a disintegrator (Unitex Engineers, Depanama, Sri Lanka). The disintegrated pairings were then dried at 70 °C using a cabinet-type dehydrator (Wessberg, Martin, Germany) for 8 h. Dried CT of each cultivar was subjected to cold press oil extraction using a micro oil expeller (Komet DD85 machine, Mönchengladbach, Germany). The remaining partially defatted CT (less than 15 % oil content) was further ground into a fine powder to make coconut testa flour (CTF) using a general-purpose grinder. Samples of CTF obtained from individual cultivars were stored at refrigerated conditions (4 °C) for further analysis. All chemicals used in this study were of analytical grade unless otherwise specified.

### 2.2 Preparation of crude polysaccharide

The crude polysaccharide (CP) fraction of CTF was extracted in accordance with the method described by Mohd Nor et al. [10] and Al-Sheraji et al. [11]. Approximately 70 g of CTF of individual cultivars was defatted by soaking in 5 times hexane for 48 h. The defatted residue of CTF was extracted at 80 °C with 500 mL of distilled water for 2 h; the procedure was repeated three times, and extracts were combined. The collected extract was concentrated in a rotary evaporator until it became 1/5 of its original volume. The concentrated extract was deproteinized with trichloroacetic. Briefly, the extract was adjusted to 3 pH using 10 % trichloroacetic acid and kept at room temperature overnight. The obtained turbid solution was centrifuged at 3075×*g* for 10 min. The precipitated protein fraction was removed by filtration, and the supernatant was collected. Subsequently, the supernatant was dialyzed against distilled water for 24 h at room temperature using 12–14 kDa dialysis bags. The dialyzed portion was filtered using Whatman no.1 filter paper and concentrated in a rotary evaporator until it became 1/5 of its original volume. Next, five times ethanol (85 %) was added to the concentrated dialyzed portion and kept for 18 h at 4 °C to allow the supernatant to drain out. The remaining mixture was centrifuged at 5432×*g* for 10 min at room temperature. The precipitate collected was oven-dried at 80 °C overnight and ground manually using a motor and pestle. The total carbohydrate content of each sample was analyzed using the phenol-sulfuric method [12]. The remaining CP fraction was kept in storage at −18 °C to perform further analyses.

### 2.3 FTIR measurements and spectral analysis

FTIR analysis of the CPs was done adopting the KBr pallet method where each sample was mixed with KBr (FT-IR grade, ≥99 % trace metals basis, Sigma Aldrich) at a 1:90 ratio and pressed into a pallet [13]. FTIR spectra were collected in the mid-infrared region of 4000–500 cm^−1^ by co-adding at 64 scans, with a resolution of 8 cm^−1^ using an FTIR Nicolet iS50 spectrometer (Thermo Nicolet, Madison, WI, USA) equipped with deuterated triglycine sulfate (DTGS) KBr detector and KBr beam splitter. Spectra were recorded as absorbance values at each data point in 4 replicates.

The manufacturer's software (OMNIC operating system, version 7.0 Thermo Nicolet, Waltham, MA, USA) program was used to pre-process the raw spectra and to obtain the peak parameters. The raw spectra were baseline-corrected and subjected to scale normalization. The resulting spectra were finally used for the identification of important spectral bands that appeared within 4000-700 cm^−1^.

## 3 Rate of hydrolysis of CPs

### 3.1 Preparation of artificial human gastric juice

The artificial human gastric juice was prepared in accordance with the method described by Abbasiliasi et al. [12]. A 8 g portion of NaCl, 0.2 g of KCl, 14.35 g of NaHPO_4_, 8.25 g of Na_2_HPO_4_.2H_2_O, 0.18 g of MgCl_2_.6H_2_O and 0.1 g of CaCl_2_.2H_2_O were dissolved in distilled water and topped up to 1000 mL. After that, a series of solutions with different acidities from pH 1, 2, 3, 4, and 5 were prepared using a 6 M HCl stock solution.

### 3.2 Degree of hydrolysis of CPs

The degree of hydrolysis of CP was determined in accordance with the method described by Abbasiliasi et al. [12] with slight modifications. Initially, a 1 % (w/v) solution of CP was prepared in distilled water. A 3 mL aliquot of artificial human gastric juice at each pH (1, 2, 3, 4, and 5) was mixed with 3 mL of CP solution, and the mixture was incubated at 37 °C in a water bath for 6 h. A constant amount was collected from each solution at time intervals of 0.5, 1, 2, 4, and 6 h. The contents of total sugar and reduced sugar were determined using phenol-sulfuric and DNSA methods, respectively. The degree of hydrolysis of each solution was calculated using the information given in Equation (1). FOS (from chicory, ≥90 %, degree of polymerization of >10) and inulin (from chicory root) were used as positive controls of this assay.

(1) $$\text{Degree}\;\text{of}\;\text{hydrolysis}=\frac{\text{Reducing}\;\text{Sugar}\;\text{released}*\;}{\text{Total}\;\text{sugar}\;\text{content}\;–\;\text{Initial}\;\text{reducing}\;\text{sugar}\;\text{content}}\times 100$$

*Reducing sugar released = Final reducing sugar content - Initial reducing sugar content.

## 4 The proliferation of probiotics on CPs

### 4.1 Bacterial culture preparation

*Lactobacillus* sp. containing yogurt culture and *Escherichia coli* were used as bacteria strains for the assay. Activated bacteria inoculum was prepared in accordance with the method described by Huebner et al. [14]. One mg of freeze-yogurt culture was grown in 10 mL of Lactobacillus MRS broth, followed by incubation for 48 h at 37 °C in anaerobic conditions. An anaerobic environment was generated using Anaerobic Gas Generator sachets (AnaeroGen™ 2.5 L, Thermo Scientific, Waltham, MA USA) inside an airtight container. To grow *E. coli*, one colony of *E. coli* was picked from a frozen culture and transferred to 10 mL of Tryptic soy broth and incubated at 37 °C overnight. One percent of incubated Tryptic Soy broth culture was transferred to M9 minimal medium and incubated at 37 °C overnight.

### 4.2 Fermentation of substrate

Test tubes containing 9.9 mL peptone water were added with 100 μL of activated bacteria inoculum and 0.1 g (1 % w/v) of each carbon source. Inulin and fructo-oligosaccharide (FOS) were used as positive controls, and the medium without a carbon source was taken as the blank. Cultures were incubated for 24 h at 37 °C.

### 4.3 Plating

*Lactobacillus* sp. and *E. coli* were plated in Lactobacillus MRS Agar and MacConkey agar, respectively. Distilled water was used to prepare serial dilution. Dilutions of each strain were selected as in the following scheme, and 40 μL of respective dilution was inoculated on agar plates in triplicates. Plates were incubated for 24 h at 37 °C.

### 4.4 Calculation of microbial proliferation and prebiotic activity score

Plates with bacterial colonies between 25 and 250 were selected for the calculation of Colony Forming Units (CFU). The cell density of each bacteria was calculated using Equation (2).

(2) $$\text{CFU}/\text{mL}=\frac{\text{Number}\;\text{of}\;\text{colonies}}{\text{Dilution}\;\text{factor}\times \text{Inoculated}\;\text{volume}\;}$$

The colony density obtained as CFU/mL was then converted to log (CFU/mL). Calculation of the growth rate (μ) and mean doubling time (T_d_) of each microorganism was done as given in Equation (3) and Equation (4), respectively, following the method described by Mansouri et al. [15].

(3) $$\mu =\frac{{\text{In}\;x}_{2}‐\text{In}\;x_{1}}{t_{2}{‐t}_{1}}$$

(4) $$\text{Td}=\frac{\text{In}\;2}{\;\mu }$$

Where x_2_ [Log (CFU mL^−1^)] is the cell density of microorganisms at time t_2_ (24 h) and x_1_ [Log (CFU mL^−1^)] is the cell density of microorganisms at time t_1_ (0 h).

The calculation of the prebiotic activity score was done as seen in Equation (5), following the method described by Huebner et al. [14].

(5) $$\text{Prebiotic}\;\text{activity}\;\text{score}=\left(\frac{\begin{array}{c} \text{Growth}\;\text{enhancement}\;\\ \text{of}\;\text{probiotic}\;\\ \text{after}\;24\;\text{hours}\;\text{on}\;\text{prebiotic}\; \end{array}}{\begin{array}{c} \text{Growth}\;\text{enhancement}\;\\ \text{of}\;\text{probiotic}\;\\ \text{after}\;24\;h\;\text{on}\;\text{glucose}\; \end{array}}\right)‐\left(\frac{\begin{array}{c} \text{Growth}\;\text{enhancement}\;\\ \text{of}\;E.\;coli\\ \text{after}\;24\;\text{hours}\;\text{on}\;\text{prebiotic} \end{array}}{\begin{array}{c} \text{Growth}\;\text{enhancement}\;\\ \text{of}\;E.\;coli\\ \text{after}\;24\;h\;\text{on}\;\text{glucose} \end{array}}\right)$$

### 4.5 Statistical analysis

All data were taken in triplicates in this study (n = 3), and the results were presented as mean ± standard deviation (SD). The experimental results of the study were analyzed using one-way ANOVA with the Minitab Software Version 20 package. When the F values were significant (p < 0.05), mean differences were compared using Tukey's test at a 5 % significance level. The manufacturer's software (OMNIC operating system, version 7.0 Thermo Nicolet) program was used for spectral pre-processing and qualitative analysis.

## 5 Results and discussion

### 5.1 Total carbohydrate contents of CPs

The yield of CPs was approximately 0.91–1.24 %, where the highest and lowest yields were given by COM and SR, respectively. The total carbohydrate contents of the CPs of cultivars varied within the range of 48.24 ± 2.70 to 60.48 ± 1.31 %. The CP extracted from TT exhibited the highest carbohydrate content, which was significantly different (p < 0.05). The COM cultivars had the lowest carbohydrate content, which was not significant (p > 0.05) in the value obtained for GT. The carbohydrate contents of SR and RT cultivars were 57.66 ± 0.78 % and 55.32 ± 0.24 %, respectively, and were found to be significantly (p < 0.05) different from the values of other cultivars. In a prior study, Abbasiliasi et al. [12] reported the total carbohydrate contents of soluble polysaccharides extracted from coconut kernel cake, which ranged from 42.56 ± 0.64 % to 55.36 ± 0.33 %. They further emphasized the impact of extraction solvent on the total carbohydrate content of the soluble polysaccharide fraction.

### 5.2 Spectral analysis of CPs

**Fig. 1:**
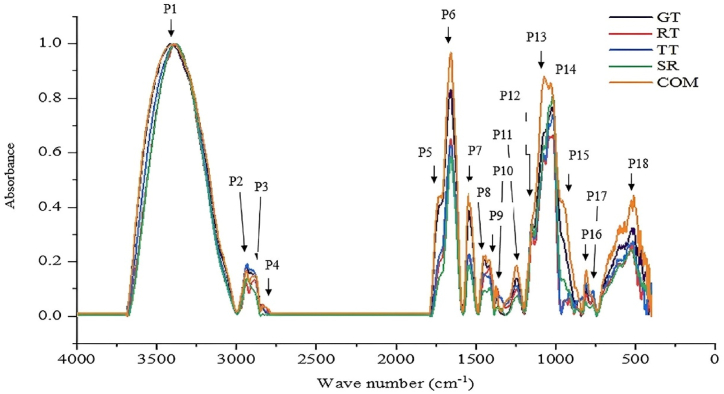
FTIR spectral overlay of crude polysaccharides extracted from coconut testa flour of different coconut cultivars. Abbreviations: GT, Gon Thembili; RT, Ran Thembili; TT, TallxTall; SR, San Ramon; COM, Commercial Hybrid.

FTIR spectra of different samples of CPs are depicted in Fig. 1. The spectral overlay indicated that carbohydrate-like substances were the major macro-molecules present in the samples. In all spectra, robust and broad absorption P1 in the range of 3350–3,405 cm^−1^ confirmed the stretching vibration of O─H groups [15]. The weak-low intensity peaks (P1 and P2) at ⁓2935 cm^−1^ (P2) and ⁓2892 cm^−1^ (P3) were attributed to the asymmetrical and symmetrical C─H stretching of methylene groups, respectively [16]. These two peaks are used to appear dominant in the majority of the oils and fats owing to the presence of aliphatic chains with multiple CH_2_ units. As CPs of this study were prepared through hot-water extraction of defatted CTF samples, there appeared to be a low abundance of lipids in CPs. This could be the main reason that the two peaks at ⁓2935 cm^−1^ (P2) and ⁓2892 cm^−1^ (P3) have become weak-low intensity peaks. The evidence in support of the presence of β-bonded polysaccharides in all CPs had surfaced through a number of spectral bands. The occurrence of peaks P13 and P14 in all CPs is a characteristic feature, indicating the presence of glycosidic bonds. Previously, Huojiaaihemaiti et al. [17] stated that strong bands between 1200 cm^−1^ – 1000 cm^−1^ could be caused by stretching vibrations of C–O side groups and C–*O*–C glycosidic bonds, which were generally indicative of the presence of glycosidic bonds. This has been affirmed by Lamarood and Ralegankar [18], who also stated that the peak around ⁓1150 cm^−1^ was generally indicative of the presence of glycosidic bonds. In their FTIR characterization of crude polysaccharides of marine algae, Fernando et al. [19] assigned the peak around ⁓1148 cm^−1^, which was most similar to P13 to glycosidic bonds. The occurrence of another peak at ⁓1075 cm^−1^ (P14) in CPs of all cultivars could also be assigned to β-glucan, which falls under the category of β-bonded polysaccharides [20]. This is in conformity with Ayimbila and Keawsompong [21], who assigned the peak at ⁓1075 cm^−1^ to β-glucan with pyranose sugars as occurred in the FTIR spectrum of CP extracted from fruiting bodies of *Lentinus squarrosulus*. All these affirmed the occurrence of β-bonded polysaccharides in the CP of all cultivars. Other than these, the occurrence of two bands at 843.2 and 893.8 cm^−1^ further confirms the presence of glycosidic bonds and carbohydrates in the CP of cultivars. They indicated the presence of sugar units, which were commonly linked by both alph- and beta-glycosidic bonds.

### 5.3 Degree of hydrolysis of crude polysaccharides

**Fig. 2:**
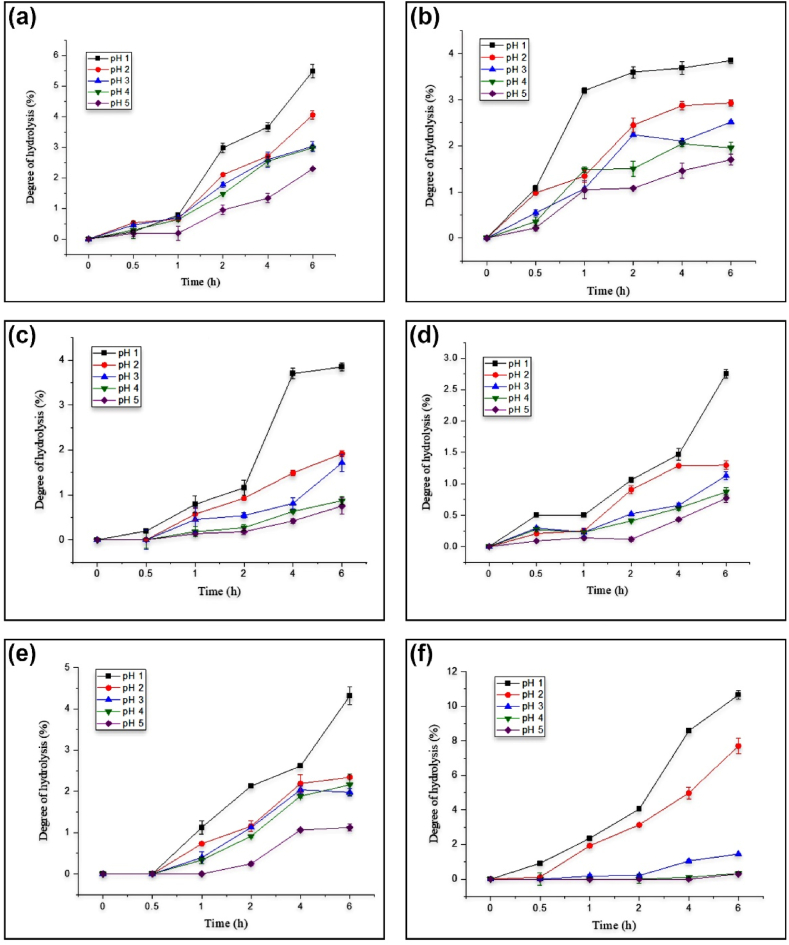
Degree of hydrolysis of crude polysaccharides obtained from coconut testa flour of different cultivars Gon Thembili (a), Ran Thembili (b), San Ramon (c), TallxTall (d), Commercial Hybrid (e), and Inulin (f) at different pH levels of 1, 2, 3,4, and 5.

Human gastric juice is actually an acidic secretion formed within the stomach lining. Its acidity is usually varied between pH 2–4 depending on the conditions inside the stomach. As the digestion process might take time, the food consumed would remain inside the stomach for about 2–4 h [10]. According to prebiotic requirements, the potential substrates should be able to resist the pH of the gastric juice so that they are not fully hydrolyzed within the period before passing on to the small intestine. The patterns of hydrolysis of CPs of coconut cultivars are depicted in Fig. 2. The overall results clearly showed the effects of pH and incubation time on the degree of hydrolysis of CP obtained from individual cultivars. In general, the degree of hydrolysis of all CPs was high at low pH but tended to decline with increasing pH. When the incubation time was prolonged, the degree of hydrolysis of each carbon source at each pH (i.e., pH = 1, 2, 3, 4, and 5) gradually increased; the longer the time of incubation, the greater the number of polysaccharides degraded to mono- and di-saccharides [10]. This could probably be due to the fact that the glycosidic bonds of CP are more easily broken down at low pH, promoting the partial hydrolysis of CPs [9,21]. Inulin, which is the positive control, also displayed a higher degree of hydrolysis at lower pH but eventually decreased when the pH was gradually increased (Fig. 2). It could be noted that the degree of hydrolysis of CP of individual cultivars was significantly (p < 0.05) lower than that of the positive control (inulin).

Among the different cultivars used in this study, the highest degree of hydrolysis was observed for GT (p < 0.05) at every pH, while the lowest was shown by TT (p < 0.05). The highest degree of hydrolysis shown by GT was 5.48 %, while the lowest degree of hydrolysis shown by TT was 2.75 %. The degree of hydrolysis of the rest of the cultivars, namely RT, SR, and COM, were 3.85 %, 3.86 %, and 4.31 %, respectively. As a notable feature, no significant (p > 0.05) difference was noticed between the degree of hydrolysis of RT and SR. These results affirmed that the CP extracted from CTF was highly resistant to gastric juice, which ranged between 94 and 97 %. Since the substrate did not fully hydrolyze in the human gastric juice, the extracted CP fulfilled the first criteria of prebiotic categorization. This was in accordance with the findings previously reported for crude polysaccharides extracted from defatted coconut kernel cake [12], defatted coconut residue [10], and defatted *Mangifera pajang* fibrous pulp [11]. In all these studies, the degree of hydrolysis of the substrates negatively correlated with the pH of the gastric juice. Concurrently, the rate of hydrolysis of their substrates increased upon a prolonged incubation time period. It should be noted that the resistance of crude polysaccharides extracted from coconut residue crude to gastric juice (⁓88 %) was lower than that of FOS (⁓98 %) [10] while that of CP extracted from coconut kernel cake (⁓98 %–99 %) was nearly similar to the resistance of FOS (⁓98 %) [12]. In our study, however, the resistance exhibited by CPs to gastric juice was relatively higher than that of inulin (⁓89 %) and FOS (⁓88 %).

### 5.4 Effect of CPs on proliferation of *Lactobacillus* sp

The calculated Colony Forming Units (CFU) of *Lactobacillus* sp. and *E. coli* are shown in Table 1. Bacterial samples that were allowed to grow in media with inulin/FOS were positive controls, while those allowed to grow in the media without any carbon source were taken as blank. Results in Table 1 showed that *Lactobacillus* sp. could use carbon sources for their proliferation since the CFU value of each carbon source was higher than that of the blank sample. The proliferation of probiotics in the presence of CP could be probably due to their solubility. As stated in previous reports, probiotics could use water-soluble carbohydrates readily, completely, and rapidly [10,22]. The molecular weight of the used polysaccharides could affect the absorption by the probiotic bacteria [23,24]. According to previous studies, probiotic growth is usually influenced by the degree of polymerization, monomeric composition, and the type of glycosidic linkages present in polysaccharides and oligosaccharides [25]. Other than these, the growth of microbes in a medium is also dependent on their ability to absorb nutrients available in the medium [26].

**Table 1:** Summary of microbial counts of *Lactobacillus* sp. and *E. coli*.

Sample	*Lactobacillus* sp. log CFU mL^−1^	*E. coli.* log CFU mL^−1^
Blank	0.00 ± 0.00^f^	7.54 ± 0.08^d^
Glucose	7.92 ± 0.00^e^	8.50 ± 0.04^b^
Inulin	8.19 ± 0.01^bc^	8.54 ± 0.04^b^
FOS	8.29 ± 0.01^a^	8.66 ± 0.03^ab^
RT	8.06 ± 0.05^d^	8.75 ± 0.06^a^
GT	8.14 ± 0.00^c^	8.53 ± 0.05^b^
TT	8.19 ± 0.01^bc^	8.31 ± 0.06^c^
SR	8.16 ± 0.01^bc^	8.57 ± 0.01^ab^
COM	8.23 ± 0.01^ab^	8.68 ± 0.04^ab^

Each value in the table represents the mean of three replicates. Means within the column that do.

not share a similar superscript are significantly different at 95 % confidence (α = 0.05).

Abbreviations: FOS, Fructooligosaccharide; COM, Commercial hybrid; GT, Gon Thembili; RT, Ran Thembili; SR.

San Ramon; TT, Tall × Tall.

Based on the details given in Table 1, among all media, the highest colony density of *Lactobacillus* sp. was observed in FOS, which was 8.29 ± 0.01 Log CFU mL^−1^. This could be because FOS could enhance the growth of Lactic acid bacteria effectively. Consequently, this could lead to increased production of butyrate and lactate, which are beneficial for gut health [27]. Among cultivars, the highest colony density of *Lactobacillus* sp. was observed for COM (8.23 ± 0.01 log CFU mL^−1^), while the lowest was observed for RT (8.06 ± 0.05 Log CFU mL^−1^). The colony density of *Lactobacillus* sp. on CP of the rest of the cultivars varied between these two extremes. Although their differences in proliferation were not significant (p > 0.05), they were significantly (p < 0.05) different from the proliferation of RT. However, no significant (p > 0.05) difference was noticed in the proliferation of *Lactobacillus* sp. in media with CP of COM and FOS/inulin. This indicated that both COM and FOS/inulin had similar effects on the proliferation of *Lactobacillus* sp. However, the growth of *Lactobacillus* sp. on media with CP of RT, GT, TT, and SR were significantly (p < 0.05) lower than the value observed for FOS. Likewise, no significant (p > 0.05) difference was observed between the proliferation of inulin and the cultivars, namely, GT, TT, and SR. Hence, it can be said that GT, TT, and SR have similar effects on *Lactobacillus* sp. proliferation similar to that of inulin. The data given in Table 1 show that the effect shown by RT could be lower on *Lactobacillus* sp. proliferation than positive controls. For instance, the proliferation of this bacteria on RT is lower than that of either inulin or FOS. Furthermore, the colony density of *Lactobacillus* sp. on glucose was significantly (p < 0.05) (7.92 ± 0.00 log CFU mL^−1^) lower than those of CP, FOS, or inulin. The proliferation of *Lactobacillus* sp. on blank media was 0.00 ± 0.00 log CFU mL^−1^ and was notably lower than CP of all cultivars, FOS, inulin, or glucose. According to a previous study by Mohd Nor et al. [10], the proliferation of *Lactobacillus. casei Shirota* and *Lactobacillus bulgaricus* in media with defatted coconut residue (DCR) was lower than that of media with inulin. The results of the present study were somewhat compatible with the CPs of most coconut cultivars except COM. It can be noted that the colony density of *Lactobacillus. casei Shirota* in the medium containing 1 % DCR was roughly similar to that of the colony density of *Lactobacillus* sp. Grown in media containing CPs of all cultivars.

### 5.5 Effects of CPs on the proliferation of pathogenic bacteria

The colony densities of *E. coli* in the medium containing CP of different cultivars were compared to those of the controls and blank, as shown in Table 1. As a matter of fact, specific activation of probiotic bacteria should occur along with the inhibition of the growth of pathogenic bacteria. The general observations suggest that the proliferation of *E. coli* was roughly similar to the pattern of *Lactobacillus* sp. The values varied between 8.31 ± 0.06 Log CFU mL^−1^ and 8.75 ± 0.06 Log CFU mL^−1^, where the highest and the lowest colony densities were recorded for RT and TT, respectively. Colony densities obtained for all cultivars except TT were not significantly (p > 0.05) different. Meanwhile, the growth of *E. coli* on media with FOS and inulin did not show any significant (p > 0.05) differences. When comparing to FOS, RT, SR, or COM did not show any significant (p > 0.05) difference with regard to the growth of *E. coli*. Growth of this bacteria on GT, SR, and COM were similar (p > 0.05) to that observed on inulin. These suggest that the effect of CPs of RT, GT, SR, and COM were similar (p > 0.05) in growth when compared to those of both positive controls. However, the proliferation of *E. coli* on TT was significantly lower than any other carbon source employed in this study. Thus, it can be predicted that CP of TT is a better inhibitor of *E. coli* than any other carbon source. Except for RT and TT, no significant (p > 0.05) difference was seen between the growth of *E. coli* on glucose and other carbon sources such as CPs of cultivars, FOS, and inulin. However, the proliferation of the blank was notably lower when compared to those of any other media employed in this study.

The growth rate of a bacteria denotes the increase in the number of bacteria within a unit of time, while the mean doubling time denotes the time required for bacteria to double their cell. It is essential to determine both the mean doubling time and growth rate of bacteria to identify the increase in cell density on a culture. The results of the growth rate and mean doubling time of *Lactobacillus* sp. and *E. coli* are depicted in Table 2. The growth rate of *Lactobacillus* sp. in CP of individual cultivars was about 0.34 Log CFU mL^−1^ h^−1^ (Table 2), but no significant (p > 0.05) difference was observed among them. The growth rate of the bacteria on CPs of all cultivars was significantly (p < 0.05) lower than that of FOS. However, the growth rate of this prebiotic substrate in media with CP of all cultivars was similar (p > 0.05) to that of inulin. Furthermore, the growth rate of FOS and inulin did not show any significant (p > 0.05) differences. Thus, it can be stated that CPs of all cultivars and positive controls have similar effects on the growth rate of *Lactobacillus* sp. According to Table 2, the growth rate of probiotics in media with glucose was significantly lower (p < 0.05) than those of other carbon sources used in this study. In addition, a notably lower growth rate of the probiotic in blank media was recorded when compared to any other carbon sources.

**Table 2:** Growth rate and mean doubling time of bacteria.

Sample	*Lactobacillus* sp.		*E*. *coli*	
	μ Log CFU mL^−1^ h^−1^	T_d_ (h)	μ Log CFU mL^−1^ h^−1^	T_d_ (h)
Blank	0.00 ± 0.00^c^	0.00 ± 0.00^c^	0.33 ± 0.01^b^	0.96 ± 0.02^a^
Glucose	0.33 ± 0.00^b^	0.91 ± 0.00^a^	0.36 ± 0.01^a^	0.85 ± 0.01^b^
Inulin	0.34 ± 0.00^ab^	0.89 ± 0.00^a^	0.36 ± 0.01^a^	0.85 ± 0.01^b^
FOS	0.35 ± 0.00^a^	0.86 ± 0.00^b^	0.36 ± 0.00^a^	0.84 ± 0.00^b^
RT	0.335 ± 0.01^b^	0.90 ± 0.01^a^	0.37 ± 0.01^a^	0.83 ± 0.02^b^
GT	0.34 ± 0.00^ab^	0.89 ± 0.00^a^	0.36 ± 0.01^a^	0.85 ± 0.01^b^
TT	0.34 ± 0.00^ab^	0.89 ± 0.00^a^	0.35 ± 0.01^a^	0.86 ± 0.02^b^
SR	0.34 ± 0.00^ab^	0.89 ± 0.00^a^	0.36 ± 0.00^a^	0.84 ± 0.00^b^
COM	0.34 ± 0.00^ab^	89 ± 0.00^a^	0.36 ± 0.00^a^	0.84 ± 0.00^b^

Each value in the table represents the mean of three replicates. Means within the column that do not share a similar.

superscript was significantly different at 95 % confidence (α = 0.05). Abbreviations: FOS, Fructo-oligosaccharide.

COM, Commercial hybrid; GT, Gon Thembili; RT, Ran Thembili; SR, San Ramon; TT, Tall × Tall; μ, Growth rate.

T_d,_ Mean doubling time.

When considering the growth rate of *E. coli* on CP of each cultivar, the rates varied between 0.35 Log CFU mL^−1^ h^−1^ and 0.37 Log CFU mL^−1^ h^−1^, where the highest and lowest were observed for RT and TT, respectively. However, no significant (p > 0.05) difference was noticed in the growth rate of the bacteria among different cultivars or between cultivars and FOS/inulin. The growth rates of *E. coli* in blank media were significantly (p < 0.05) less than those of all cultivars.

When calculating the mean doubling time, similar results were noticed for both bacteria in each media (Table 2). The mean doubling time of *Lactobacillus* sp. on CP of all cultivars observed was between 0.89 and 0.90 h, where the highest was observed for RT. The mean doubling time of *Lactobacillus* sp. on inulin did not show a significant (p > 0.05) difference when compared to those of CP of all cultivars. Nevertheless, the mean doubling time of FOS was significantly (p < 0.05) different from those of all cultivars. Moreover, the mean doubling time of *Lactobacillus* sp. on blank media was notably lower than those of all tested carbon sources in this study. As shown in Table 2, the mean doubling times of *E. coli* on CP of individual cultivars ranged from 0.83 to 0.86 h, where the highest and lowest were exhibited by TT and RT, respectively. However, no significant (p > 0.05) difference was observed among the mean doubling times of this bacteria on the rest of the cultivars. When compared to the mean doubling time of FOS/inulin, the same CP of all cultivars did not show any significant (p > 0.05) difference. However, the mean doubling times of *E. coli* on blank media was 0.96 ± 0.02 h, which was significantly higher (p < 0.05) than those of the media containing other carbon sources, including CP of all cultivars, FOS, inulin, and glucose. Hence, it can be suggested that the presence of carbon sources reduces the mean doubling time of *E. coli* bacteria.

#### 5.5.1 Prebiotic activity scores

The data presented in Table 3 show the prebiotic activity scores to identify the prebiotic potential of the different carbon sources. The method adopted is based on comparing the growth of probiotics and *E. coli* like gastrointestinal strains. This would select the combined effect of probiotics and prebiotics in food, predicting the extent of prebiotic activity [28]. The calculated prebiotic activity score corresponding to each carbon source relevant to *Lactobacillus* sp. was between 0.50 and 1.09 (Table 3), where the highest value was observed for CP of SR and the lowest was observed for CP of COM. Based on this, it can be stated that SR had the highest prebiotic effect while COM had the least prebiotic effect. The prebiotic activity scores of CP of most coconut cultivars except SR were comparatively lower than those of either inulin or FOS. However, the prebiotic activity score of CP of SR was roughly similar to that of FOS. Since the prebiotic activity scores of the CP of all cultivars are higher than 0.5, it can be stated that the CP of all cultivars could have the capacity as prebiotics in food products [29].

**Table 3:** Prebiotic activity score of individual carbon source.

Carbon source	Prebiotic activity score (*Lactobacillus* sp.*)*
Inulin	0.81
FOS	0.92
RT	0.73
GT	0.62
TT	0.71
SR	1.09
COM	0.50

Abbreviations: FOS, Fructooligosaccharide; COM, Commercial hybrid; GT, Gon Thembili; RT, Ran Thembili; SR, San Ramon; TT, Tall × Tall.

## 6 Conclusions

FTIR characterization showed that the hot-water extraction adopted in this study was successful in recovering CPs as substrates for prebiotic study. Although the hydroxyl group of CP was the main functional group of the CPs, the FTIR peaks occurring at ⁓1148 cm^−1^ and ⁓1075 cm^−1^ were indicative of the presence of β-glucan while peak at 1244 cm^−1^ confirmed the presence of β-1,4 glycosidic bonds. Based on the hydrolysis test results, most of the CPs displayed high resistance to human stomach conditions (94 %–97 % resistance). The GT cultivar displayed the highest resistance for hydrolysis, while the TT cultivar showed the lowest. CP of all cultivars was able to stimulate *Lactobacillus* sp. probiotic to proliferate. The highest colony density of *Lactobacillus* sp. after 24 h of fermentation was observed on media with COM (8.23 ± 0.01 Log CFU mL^−1^), while the lowest was on media with RT (8.06 ± 0.05 Log CFU mL^−1^). The mean doubling time of *Lactobacillus* sp. on media with CP of different cultivars was similar (p > 0.05). The prebiotic activity score of CPs of all cultivars was higher than 0.50. All these suggest that CPs of all cultivars could be fermented by prebiotics, which can stimulate the growth of *Lactobacillus* sp. The overall findings suggest the potential value of CTF as an emerging source of functional food. This study exclaimed the prebiotic characteristics through in-vitro studies, but this approach may have limitations in obtaining comprehensive observations. Therefore, more extensive animal and clinical studies are needed to evaluate the prebiotic effect of CTF and understand its underlying mechanism. However, this study could serve as a valuable starting point for further research in this area, providing insights for future studies.

## Funding

None.

## Data availability statement

The datasets generated for this study will be made available via reasonable request to the corresponding author.

## CRediT authorship contribution statement

**Rasika Gunarathne:** Writing – original draft, Methodology, Formal analysis, Data curation, Conceptualization. **Sandani Wijenayake:** Validation, Methodology, Formal analysis, Data curation. **Chandhi Yalegama:** Writing – review & editing, Supervision, Funding acquisition, Conceptualization. **Nazrim Mohammed Marikkar:** Validation, Supervision, Resources, Funding acquisition. **Jun Lu:** Writing – review & editing, Supervision, Investigation, Funding acquisition.

## Declaration of competing interest

The authors declare the following financial interests/personal relationships which may be considered as potential competing interests:The corresponding author is an Associate Editor of Heliyon - J.L. If there are other authors, they declare that they have no known competing financial interests or personal relationships that could have appeared to influence the work reported in this paper.
